# The effects of high dose and highly fractionated radiation on distraction osteogenesis in the murine mandible

**DOI:** 10.1186/1748-717X-7-151

**Published:** 2012-09-07

**Authors:** Laura A Monson, Christi M Cavaliere, Sagar S Deshpande, Alexander L Ayzengart, Steven R Buchman

**Affiliations:** 1 Craniofacial Research Laboratory, University of Michigan, 2228 BSRB, 109 Zina Pitcher, Ann Arbor, 48109, USA

## Abstract

The ability of irradiated tissue to support bony growth remains poorly defined, although there are anecdotal cases reported showing mixed results for the use of mandibular distraction osteogenesis after radiation for head and neck cancer. Many of these reports lack objective measures that would allow adequate analysis of outcomes or efficacy. The purpose of this experiment was to utilize a rat model of mandibular distraction osteogenesis after high dose and highly fractionated radiation therapy and to evaluate and quantify distracted bone formation under these conditions. Male Sprague–Dawley rats underwent 12 fractions of external beam radiation (48 Gray) of the left mandible. Following a two week recovery period, an external frame distractor was applied and gradual distraction of the mandible was performed. Tissue was harvested after a twenty-eight day consolidation period. Gross, radiologic and histological evaluations were undertaken. Those animals subjected to pre-operative radiation showed severe attenuation of bone formation including bone atrophy, incomplete bridging of the distraction gap, and gross bony defects or non-union. Although physical lengthening was achieved, the irradiated bone consistently demonstrated marked damaging effects on the normal process of distraction osteogenesis. This murine model has provided reliable evidence of the injurious effects of high dose radiation on bone repair and regeneration in distraction osteogenesis utilizing accurate and reproducible metrics. These results can now be used to assist in the development of therapies directed at mitigating the adverse consequences of radiation on the regeneration of bone and to optimize distraction osteogenesis so it can be successfully applied to post-oncologic reconstruction.

## Introduction

The ability of irradiated tissues to support the process of distraction osteogenesis (DO) after clinically relevant doses of external beam radiation is unknown. Anecdotal case reports in the literature have shown mixed results, and animal studies have been small and inconclusive lacking objective measures that would allow adequate analysis of outcomes or efficacy. Radiation therapy is detrimental to bone and soft tissue healing as well as to normal bone remodeling. Negative effects include poor fracture healing [1-3], impaired growth [4], decreased mechanical strength [5,6], and bone atrophy due to increased bone resorption and reduced formation. Irradiated bone undergoes a loss of bone cells and fibrosis. Radiation disrupts bone microvasculature causing decreased vascular density and obliteration of small blood vessels that progressively worsens over time [7]. Recovery of irradiated bone is usually poor and late complications such as osteoradionecrosis can be devastating.

Head and neck cancer (HNC) affects 500,000 people worldwide each year and 52,000 in the United States alone. Many of these patients will require multimodality treatment with surgery, radiation, and chemotherapy. Although radiotherapy has increased survival, it also results in damage to adjacent normal tissues leading to significant morbidity [8-10]. The corrosive impact of these radiation induced side effects can be unrelenting and their complex management is rarely straightforward. Surgical treatment of HNC poses an ongoing challenge as it is complicated by the severely problematic wound healing issues consequent to adjuvant radiation therapy [11-17]. Standard of care currently dictates mandibular reconstruction utilizing free tissue transfer, requiring the harvest of bone and tissue from other parts of the body (leg, rib, scapula, or iliac crest).

Advantages of microvascular reconstruction include a rich blood supply, transfer of healthy composite flaps of bone and soft tissue that have not been subject to irradiation [18], and a high rate of successful wound closure. Disadvantages include long operative time, significant technical demands, donor site morbidity and the occasional need for two flaps to achieve adequate bone and soft tissue coverage. The significant risks associated with free tissue transfer often exclude their use in both the elderly and the infirm. Perhaps the most troubling clinical consequence of free tissue transfer is that commonly associated wound healing complications can force delays in the initiation of adjuvant therapy jeopardizing prognosis as well as quality of life. A less invasive reconstructive method that would utilize local tissues to restore structural and functional integrity while avoiding donor site morbidity would clearly be desirable.

DO avoids donor site morbidity, generates vascularized endogenous bone and soft tissue, involves a less invasive approach with shorter operative time with the potential of a more rapid recovery and reduction of overall treatment costs. Since DO has already been widely used to treat congenital and traumatic mandibular deficiencies, application of this technique to oncologic reconstruction would be a natural extension of this powerful technology, however, the use of these tissues is currently avoided due to the detrimental effects of irradiation as well as the paucity of local substrate.

Existing animal models of DO following radiation therapy are extremely limited, and results from these animal studies are mixed and largely uncontrolled [19-21]. Anecdotal case reports concerning the use of mandibular distraction osteogenesis (MDO) in irradiated patients have had variable outcomes without long term follow-up [22-27]. Furthermore, the spectrum of radiation doses deemed necessary by radiation oncologists for the treatment of the variety of head and neck cancers adds additional uncertainty for outcomes of mandibular reconstruction. For these reasons, the role of MDO in oncologic reconstruction remains ambiguous.

The purpose of this study was to utilize a reproducible rat model of MDO to evaluate, quantify, and document the damaging effects of high dose highly fractionated radiation (XRT) on distraction induced new bone formation. Our hypothesis is that the pathologic effects of radiation on bone formation and healing will lead to a severe and measurable impairment of DO. The specific aim of this investigation is to determine the effects of high dose and highly fractionated radiation on bone formation during MDO utilizing histologic and radiographic outcome measures. The over-arching goal of our work is to generate specific metrics of diminished bone quality within the irradiated mandible and then to develop treatment strategies to assuage the adverse impact of radiation induced injury on new bone formation and healing in order to optimize reconstruction and repair.

## Methods

**Figure 1:**
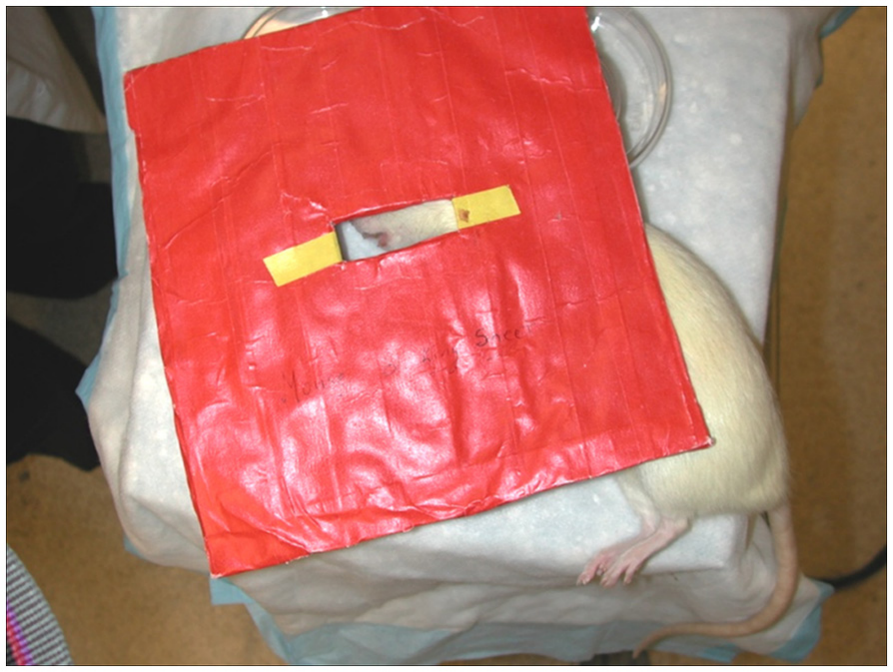
**Shielding for murine mandibular radiation therapy.** The rat was anesthetized and placed under lead shielding which only exposed the left mandible. X-rays ensured that radiation tapered off quickly and did not administer a therapeutic dose to the contralateral side.

All animal protocols were approved by the University of Michigan Committee on the Use and Care of Laboratory Animals in accordance with federal standards. Radiation was performed in collaboration with the University of Michigan (UofM) department of Radiation Oncology. Adult male Sprague–Dawley rats (375–400 grams) underwent XRT of the left mandible (control N = 12, XRT N = 12). Anesthesia included inhalational isofluorane. A lead shield was placed over the entire body with a rectangular aperture overlying the left mandible (Figure 1). The total radiation dose was 48 Gray divided in twelve daily fractions of 4 Gray (Pantak DXT Orthovoltage, 300 kV x-rays) for a total of 48 Gy. A two week recovery period was allowed between the completion of radiation therapy and operative distractor placement.

**Figure 2:**
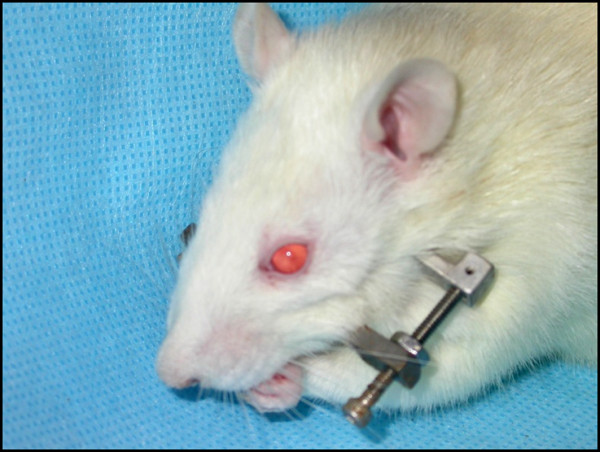
Rat with custom-built titanium bilateral fixator with unilateral distraction device.

The surgical procedure was carried out as previously described [19]. Briefly, a custom-machined external frame distraction device was placed using a midline submandibular incision (Figure 2). An osteotomy was performed posterior to the left molars and the distraction device was adjusted to re-approximate the cut bone edges. Subcutaneous fluid for hydration, chloramphenicol, and butorphanol were given twice daily through postoperative day four. Cephalexin suspension was added to the drinking water for the remainder of the postoperative period. Animals were given free access to soft moist chow. Following a four day latency period, distraction began at a rate of 0.3 mm twice per day, up to 5.1 mm. In previous studies, 5.1 mm was shown to be a critical-size defect using this model [19]. The control group underwent the same surgical procedure without pre-operative radiation. Due to development of a cross-bite, upper incisor teeth were trimmed as needed throughout the post-operative period. All animals were followed until postoperative day forty and then euthanized with intraperitoneal pentobarbital (50 mg/kg).

At the time of harvest, tissue was placed in cold 4% paraformaldehyde. Lateral radiographs of the mandible (Faxitron X-ray, Buffalo Grove, IL) were performed. Standardized settings were used for all specimens. After 72 hours of fixation, tissue was rinsed in phosphate buffered saline and transferred to a formic acid/sodium citrate decalcification solution (equal parts 40% formic acid and 6.25% sodium citrate) and kept at 4 degrees Celsius. Decalcification solution was changed every 2 to 3 days and specimens were followed radiographically for evidence of complete decalcification, approximately 14 days. Specimens were then dehydrated in 70% ethanol and embedded in paraffin. Tissue was cut into 10 micrometer capital sections and mounted on glass slides coated with 3-aminopropyl-triethoxysilane (SigmaChemical Company, St. Louis, MO).

### Radiographic evaluation

Radiographs were placed on a viewing box with a digital camera (Coolpix 4500, Nikon Inc., Melville, NY), mounted a fixed distance above the film. Camera settings were standardized and all photographs performed in a single session. Digital photographs were analyzed using an imaging software package (BIOQUANT Image Analysis Corporation, Nashville, TN). A standard rectangular template was centered over the distraction gap, with the inferior border of the rectangle aligned with the inferior mandibular border. The mean grayscale pixel density within the rectangular area was calculated and recorded for each radiograph. This scale ranges from no mineral content (zero) to full mineralization with saturated white pixels (250). Comparison of pixel density gives an estimate of the difference in mineralized distraction gap tissue between the two groups. The mean value for the control group and experimental group were calculated and statistical analysis was performed using two-tailed Students t-test with significance based on p<0.05.

### Histologic evaluation

Sections were chosen for each specimen at 100 micrometer intervals and stained with alcian blue-hematoxylin. Each section was photographed at 3.5x magnification using the same digital camera mounted on a dissecting microscope (SMZ-10A, Nikon, Inc.). Using the BIOQUANT software, a standardized region of interest was outlined over the distraction gap. The color threshold for bone was selected and the percentage of the rectangular are filled with bone was automatically calculated. This threshold captured both remodeled bone and early woven bone, giving an estimate of bone formation that would depend less on mineralization than our radiographic analysis. The section with the greatest percentage bone area was selected for each specimen. The mean percent bone area for each group was determined and statistical comparison performed using a two-tailed Students t-test.

## Results

During the period of irradiation, animals developed mucositis and hair loss along the left mandible. All mucositis in irradiated animals had resolved by time of surgery. Intraoperatively, irradiated bone was noted to be significantly thinner than non-irradiated bone. There were no significantly increased difficulties with distractor placement, however. Postoperatively, control and experimental animals experienced similar weight loss and maintained comparable cage activity. An increased number of irradiated animals developed superficial anterior pin site infections, compared with our previous studies. The sites were treated with topical 3% hydrogen peroxide and all infections resolved within days. Since the anterior pin site is distant from the osteotomy site, none of these animals were excluded from the study. Control and experimental animals were of similar weights by the time of sacrifice.

### Gross evaluation

**Figure 3:**
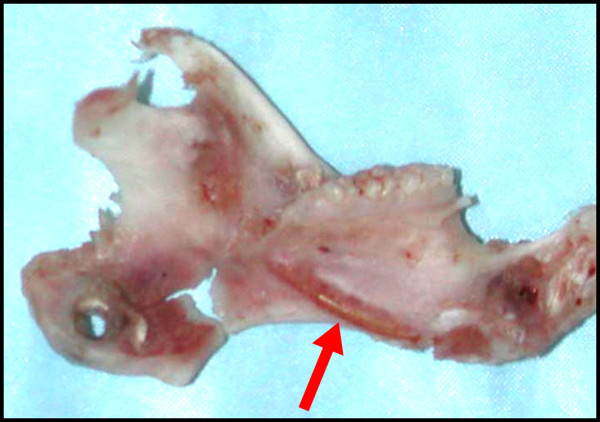
**Rat mandible following high-dose, high-fractionation radiation therapy, distraction osteogenesis, and sacrifice on post-operative day 40.** Arrow indicates the exposed incisor root. Also note the gross defects in the distracted region.

At the time of tissue harvest, irradiated bone was noted to be significantly thinner than non-irradiated bone and occasionally had gross defects. Normal appearance of the skin and muscle tissue was noted at the osteotomy site in all animals. In irradiated animals, the tongue, gingiva and molar teeth showed no evidence of lesions and no loose teeth were noted. Irradiated animals did have hair loss over the left hemi-mandible. Osseous tissue differed significantly in animals treated with pre-operative irradiation. Severe atrophic changes occurred in all irradiated specimens and the incisor was exposed along the inferior mandibular border in several irradiated animals. (Figure 3).

### Radiographic evaluation

**Figure 4:**
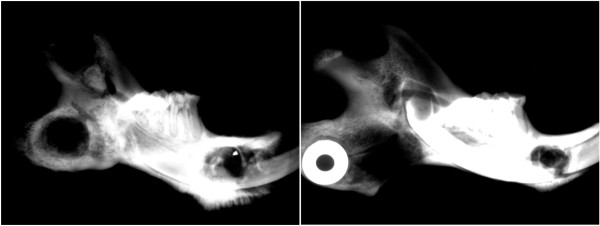
**Faxitron radiographs of control (left) and irradiated (right) mandibles that underwent distraction osteogenesis.** Note the incomplete bridging in the irradiated specimen, in contrast to the robust bridging in the control specimen.

**Figure 5:**
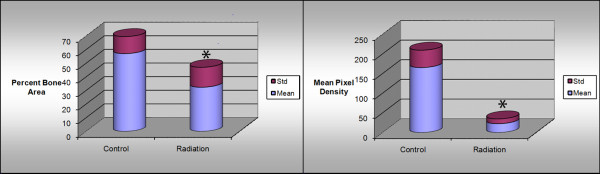
**Comparisons of histological percent bone area as well as radiological mean pixel density.** * indicates significance. Significance taken at p <0.05.

Radiographic evaluation demonstrated overall bone atrophy of irradiated specimens compared to controls (Figure 4). The distal mandibular bone segment in many specimens became severely radiolucent and demonstrated considerable resorption. The distraction gap was almost fully bridged and beginning to calcify in control specimens, but remained minimally healed in irradiated specimens. Comparison of radiographs from both groups showed a mean pixel density of 76.36 +/− 34.91 in the irradiated animals and 165.77 +/− 47.77 in controls (p <0.05), (Figure 5).

### Histologic evaluation

**Figure 6:**
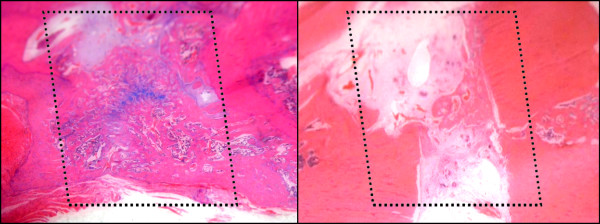
**Alcian blue-hematoxylin histological sections of control (left) and irradiated (right) mandibles that underwent distraction osteogenesis.** The regions of interest are outlined with dotted lines. Note the incomplete bridging of the distracted sample, in comparison to control. Minor chondrogenesis was observed in control (blue stain); this was not observed in irradiated samples.

In control specimens, bone fully bridges the distraction gap with trabeculae and a well-developed marrow cavity visible. The margin between native and newly formed bone is difficult to distinguish. In irradiated specimens, however, minimal bone formation occurred at the cut edges and bony bridges crossing the distraction gap are infrequent. A large cavity can be seen within the distraction gap in several specimens shows that an average of 32.41+/− 14.61% of the area is made up of bone in irradiated specimens compared to 57.07+/−12.51% in non-irradiated controls (p <0.05), (Figures 5, 6).

## Discussion

The utilization of DO for tissue replacement after oncologic resection or as a reconstructive option for deformations secondary to irradiated bone could have immense potential therapeutic ramifications. The role of DO for reconstruction of mandibular defects following therapeutic XRT, however, remains unknown. Clinical and experimental reports have been heretofore quite limited in both follow up and number and the effect of XRT on DO is still indeterminate. Previous experimental literature has been limited to inconvenient model systems such as rabbits (which are not well characterized and do not lend the model to experimental flexibility down the road) or canines (which, as large animal models, are subject to the same constraints as rabbits, with the additional burdens of being more expensive and having greater housing requirements) [20,21]. As such, this experiment utilized a rat model, which has isogenic variants for future stem cell study and abundant genetic and immunohistochemical markers, and is thus optimized for future experimentation.

This experiment demonstrates the effect of high dose, highly fractionated XRT on DO bone formation. Two weeks after the radiation period and at the time of surgery, bone grossly appeared minimally affected by the radiation. At the time of harvest, gross examination demonstrated severe bone atrophy with erosion of the bony cortex that normally surrounds the incisor and overall thinning of the mandible. Persistent bony defects were visible within the distraction gap of several irradiated specimens. These defects were not seen in any of the control animals and had not been previously seen in any prior experiments using distraction without irradiation. Radiographic evaluation further confirmed the compromised healing and mineralization of the distraction gap. Quantitative comparisons indicated a significant difference between the irradiated and non-irradiated groups. Histologic examination similarly demonstrated a significant quantitative difference in the area of bony bridging across the distraction gap as well as extensive cell death with empty lacunae and minimal new bone formation. These findings highlight the consistent detrimental effects of irradiation on the process of DO.

However, the translation between an experimental rat model and the clinic is not necessarily linear. Rat and human metabolism is significantly different, so cell growth and bone recovery rates are different at the bench as opposed to the bedside. Rat tissue has a greater susceptibility to radiation, therefore lower doses of radiation are necessary to achieve the same level of tissue damage. Finally, these rats have not experienced damage to their craniofacial skeleton prior to this experiment. The scar contraction of distraction site caused by previous surgery or chemotherapy may be detrimental to the process of distraction osteogenesis. On the other hand, the skin flaps to cover the defect after tumor ablation may work to recover the circulation of tissue envelope over previous irradiated mandible.

Utilization of DO in head and neck cancer is extremely appealing; patients could undergo large composite tissue resection and immediate soft tissue reconstruction with local flaps or microvascular free tissue transfer. Flaps could be chosen on soft tissue coverage needs alone, without the need to incorporate bone. Postoperative radiation therapy would proceed sooner as the wound healing period would be truncated. Replacement of bone through transport DO could be performed on an elective basis after completion of XRT. For elderly patients or patients in whom microvascular free tissue transfer would pose an extreme health risk, DO alone might provide a less invasive alternative. DO could also provide an additional reconstructive option after flap failure, bone resorption or osteoradionecrosis.

Over the past two decades, DO has evolved from a technique mainly for reconstruction of select cases of congenital mandibular deficiency to a frequently applied option for reconstruction of the mandible, midface and cranial vault. While its role in cases of congenital bony deficiencies and traumatic bony loss is well established, its role in head and neck reconstruction is mainly based on anecdotal case reports, both those touting successes as well as failures. The advantages of being less invasive, having a shorter operating time and avoiding donor site morbidity make it an appealing option for the head and neck cancer patient who is often a less than ideal surgical candidate. Moreover, recent research has shown DO to be an inherently vasculogenic process, thereby stimulating a hypovascular wound healing environment [28]. The complexities of the irradiated bed and the subsequent challenges to wound healing have, to this point, made microvascular free tissue transfer the gold standard as it has proven to be the most reliable reconstructive option to date. The possibility of MDO for reconstructing the head and neck cancer patient was proposed as early as 1994 [9], however the past decade has provided minimal clinical evidence of reproducible successes.

## Conclusion

As seen in this study, radiation therapy severely compromises distraction bone formation. The limitations of this experiment must be considered. The turnover rate of bone likely differs in the rat and human undergoing oncologic treatment. The time interval for recovery following radiation and the length of latency and consolidation do not directly translate to the clinical setting. Similarly, MDO in oncologic reconstruction may be performed as transport distraction, which is not possible in our small animal model. The utility of this model lies not in direct applicability to the clinical setting, but the potential to gain a better understanding of the effects of radiation on distraction bone formation at both the structural and molecular level. We conclude that DO is possible in the setting of radiation; however, there is significant potential for complications. Further work must focus on optimizing the conditions that will allow DO to be utilized as a reliable reconstructive option for the oncologic patient.

## Competing interests

The author(s) declare that they have no competing interests.

## Authors’ contributions

LAM, SSD, SRB all partook in manuscript preparation, editing, and submission. CMC and ALA carried out the experiment and handled animal care. Study design was carried out by CMC and SRB. All authors read and approved the final manuscript.
